# An Analysis of Eleven Cases of Dysentery

**Published:** 1856-08

**Authors:** Austin W. Nichols

**Affiliations:** Buffalo


### ART. II.—*An Analysis of Eleven Cases of Dysentery.*

Reported by Austin W. Nichols, M. D.

In this analysis the reporter has pursued the plan of Dr. Flint, Buff. Med. Jour., vol. ix. The subject has been divided into similar sections, most of which have been placed in tabular form; the reader, therefore, will have no difficulty in making a comparison of the two reports. Attention is invited to section 9, on the "causes," and to section 10, on the "treatment," of dysentery.

| No. | Name. | Age. | Sex. | Occupation. | Month and Year | Previous health and constitution. | Duration before com'g under ob'n |
|---|---|---|---|---|---|---|---|
| 1 | I. D. B. | 23 y. | M. | Med.Stud't | Aug., 1855. | Tons'is,int't fev. | Immediately. |
| 2 | H. B. | 15 y. | M. | Farmer. | " " | Grow'g rapidly. | " |
| 3 | A. B. | 3 y. | F. | | " " | Wean'g attem'd | " |
| 4 | Z. B. | 19 y. | M. | Farmer. | " " | Good health. | " |
| 5 | A. E. B. | 17 y. | F. | | Sept., " | Intermit. fever. | " |
| 6 | Mrs. D. | 79 y. | F. | | " " | Good constitu'n | " |
| 7 | Mr. H. | 45 y. | M. | Cooper. | Aug., " | Intermit. fever. | |
| 8 | I. S. | 47 y. | M. | Laborer. | " " | Good health. | |
| 9 | Mrs M. | 25 y. | F. | | " " | " " | Immediately. |
| 10 | V. A. | 28 y. | F. | | Aug., 1854. | " " | One day. |
| 11 | A. N. | 23 y. | M. | | Aug., 1853. | " " | |

*Synopsis of Section First.*

1. Age: Oldest, 79 years.
   Youngest, 3 years.
   3 cases over 30 years.
   8 cases under 30 years.
   $29\frac{5}{11}$ years, average age.
2. Sex: 6 males.
   5 females.
3. Occupation: 2 cases in the city.
   9 " " " country.
   4 farmers, 3 on a farm.
   All in private practice.
4. Month: 9 in August.
   2 in September.
5. Previous health: 7 good health.
   4 indifferent.

SECTION SECOND.

*Symptoms before Disease manifested itself.*

| No. | Diarrhœa. | Pain. | |
|---|---|---|---|
| 1 | Two days. | Headache. | |
| 2 | One " | None. | |
| 3 | Three " | " | |
| 4 | One " | " | |
| 5 | " " | Griping. | |
| 6 | Two " | None. | |
| 7, 8, 9 | Unknown. | (9) Griping. | |
| 10 | Three days. | None. | |
| 11 | Two hours. | " | |

*Synopsis of Section Second.*

1. Diarrhœa: 1 case, two hours.
3 cases, one day.
2 " two days.
2 " three days.
3 " unknown.

2. Pain: 1 case, in head.
2 cases, in abdomen.
3 " no pain.

SECTION THIRD.

*Symptoms referable to Digestive System.*

| No. | Appetite. | Thirst. | Tongue. | Nausea and Vomiting. | Tympanites. |
|---|---|---|---|---|---|
| 1 | Anorexia. | Great. | Coated. | Frequent vomiting. | None. |
| 2 | Good. | Moderate. | | Vomiting. | " |
| 3 | Little. | " | | None. | " |
| 4 | Good. | None. | | " | " |
| 5 | Anorexia. | Great. | Coated. | Frequent vomiting. | " |
| 6 | " | Moderate. | Reddish. | Very frequent " | Moderate. |
| 7 | Some. | | | None. | |
| 8 | " | | | " | |
| 9 | Good. | None. | | " | |
| 10 | Little. | Great. | | Nausea. | None. |
| 11 | Good. | None. | | " | " |

SECTION THIRD—CONTINUED.

| No. | Abdom. Tenderness. | Tormina. | Tenesmus. | Discharges. |
|---|---|---|---|---|
| 1 | Epigastric, mod. | Severe. | Moderate. | Mucus and blood. |
| 2 | None. | " | Slight. | " " " |
| 3 | " | Slight. | Moderate. | " " " |
| 4 | " | " | Slight. | Mucus alone |
| 5 | Iliacs moderate. | Frequent. | " | " and blood. |
| 6 | Whole abdomen. | Slight. | " | Sero-sanguin. |
| 7 | Unknown. | " | Moderate. | Mucus and blood. |
| 8 | " | " | " | " " " |
| 9 | None. | None. | " | Mucus alone. |
| 10 | Left iliac, mod. | Slight. | Considerable. | Mucus and blood, sero-sanguin. |
| 11 | None. | None. | " | Mucus and blood. |

*Synopsis of Section Third.*

1. Appetite: anorexia, in 3 cases.
little, " 4 "
good, " 4 "

2. Thirst: great, " 3 cases.
moderate, " 3 "
none, " 3 "
not stated, " 2 "
3. Tongue: coated, " 2 "
reddish, " 1 "
varying, " 8 "
4. Nausia and vomiting: nausea, in 2 cases.
vomiting, 4 "
neither, 5 "

In case, No. 5, the liquid vomited was of a dirty sea-green color, without sediment.

5. Tormina: often and severe, in 3 cases.
slight or rare, " 6 "
6. Tenesmus: considerable, " 2 "
moderate, " 5 "
slight, " 4 "
7. Discharges: mucus alone, " 2 "
mucus and blood, 9 "
sero-sanguin, in 2 "

SECTION FOURTH.

*Symptoms referable to Circulation.*

| No. | Capillary conges'n. | PULSE. In health. | Average. | Max. | Min. | Tense or Compressible. |
|---|---|---|---|---|---|---|
| 1 | None. | 72 | 108 | 114 | 88 | Compressible. |
| 2 | " | 76 | 92 | 100 | 78 | " |
| 3 | " | 85 | 100 | 112 | 90 | " |
| 4 | " | 72 | | | | At first, tense. |
| 5 | On cheeks. | 76 | 104 | 116 | 88 | Compressible. |
| 6 | " face. | | 114 | 130 | 100 | At first, tense. |
| 7 | Not known. | Unknown. | | | | Compressible. |
| 8 | " " | " | | | | " |
| 9 | On cheeks. | 76 | | | | " |
| 10 | " " | Unknown. | | 116 | | " |
| 11 | None. | 70 | | 92 | | " |

*Remarks on Section Fourth.* A moderate degree of capillary congestion existed in four cases. The number of pulsations, a week or ten days after recovery, is given, in order to show the state of the circulation in health. The pulse was tense and corded in two cases, for a few hours; but in the others, it was compressible from the first.

### SECTION FIFTH.

*Symptoms referable to Nervous System.*

Nos. 6 and 10, incoherent muttering; No. 1, mind confused; Nos. 1 and 10, headache a prominent symptom; No. 5, had a dragging sensation in the loins. Nos. 1 and 10, were troubled with jactitation.

### SECTION SIXTH.

*State of the Skin.*

Nos. 2, 3, 4, 7, 8, 9 and 11, the skin—though dry at first—soon became moist, and continued so during most of the illness. Nos. 1 and 5, the skin was dry for the greater part of the time. No. 6, the skin was cold and clammy from the first; some days, however, the skin was of its natural temperature. (Dr. Rutherford, of Harrisburg, Pa., an intelligent practitioner of thirty years experience, informs me that very few of his patients who exhibited this symptom in the beginning of the disease, recovered.) No. 10, possessed a moist skin, with perspiration, now and then, throughout the sickness.

### SECTION SEVENTH.

*Symptoms referable to Respiration.*

In case, No. 6, the respiration was frequently suspended from one to three minutes, about the fourth and fifth weeks of her illness.

### SECTION EIGHTH.

*Duration of Disease and Termination.*

*Remarks.* Only one of the eleven cases was fatal; the manner of death was by asthenia. There were no relapses. No. 5 had a previous attaek of dysentery, three or four years ago. The duration of the disease does not include the time the previous diarrhœa lasted, but is reckoned from the onset of dysenteric symptoms.

| No. | Termination. | Recovery. | Remarks. |
|---|---|---|---|
| 1 | Recovery. | 7 days. | The fatal case continued 34 days. The average duration in the other cases, was 4 days and 4 1-5 hours. |
| 2 | " | 3 " | |
| 3 | " | 4 " | |
| 4 | " | 1 " | |
| 5 | " | 6 " | |
| 6 | Death. | 34 " | |
| 7 | Recovery. | 4 " | |
| 8 | " | 8 " | |
| 9 | " | 6 " | |
| 10 | " | 4 " | |
| 11 | " | 6 hours. | |

### SECTION NINTH.

### *Causes of the Disease.*

No. 7, 8 and 9, were attacked after exposure to wet and to the cool night air; the air being sultry during day-time. No. 10 occurred in the same house where a cholera patient laid sick, and who subsequently recovered. No. 11 took place in the person of the writer, from a sudden change from the limestone water of Pennsylvania to the water from the alluvial wells near the Monongahela river, in Virginia. The remaining cases, Nos. 1, 2, 3, 4, 5 and 6, are possessed of some interest. How did they arise? They occurred in a household of thirteen persons, consisting of the parents and six children, the grandmother, two male and two female servants; all these persons ate and slept in the same house. The country is pleasant and healthy, periodical fever prevailing more or less, however, every season. This year, the fever had been quite epidemic; not only had this family been affected, but almost every person in that section had suffered from an attack of remittent, or—more frequently—intermittent fever. The dysentery arose in the month of August, the thermometer being about 80° to 90°, in the day time, and varying from 50° to 65° at night; a great quantity of rain had fallen, the storms being accompanied with chilling winds. The fruit was ripening rapidly, but was extremely juicy; this family and most all of the families, indulged, as usual, in the fruit. Of the six cases, No. 1 had had an attack of tonsillitis, terminating in profuse suppuration; Nos. 1 and 5 had frequent attacks of intermittent fever; No. 3 had been attempted to be weaned; the other members of the family were in good health and of good constitutions. Dysentery was not common at this season, nor had it been for several years previously. It was prevailing about three miles south of this house, in a tract of country the circumference of which was four or five miles. Within this space nearly every member of more than twenty families, was attacked during August and September. No communication, whatever, had been held by the above household, nor by any person in the neighborhood, with this infected district. The inhabitants there believed the disease to be contagious, and attributed the origin of the sickness to the use of the peaches and other fruit. Two cases of dysentery occurred during the early part of August, near a creek, about two miles from this house. Of the six cases, No. 1 had seen both of these persons, several times during their sickness, and the last time was only three days before he was attacked with diarrhœa, which was the precursor of his dysentery. Only two days previous to this onset of dysentery, he had assisted in the burial of a person who had died from a chronic disease of the liver. He did not remain in the apartment more than ten minutes, as the corpse was in a state of decomposition; he had neglected to change his clothes on his return home. This occurred at 7, A. M.; about 3, P. M., he was attacked with a diarrhœa which continued next day, and was not controlled by laudanum taken the first evening; the next day he took ten grains of calomel, which operated by evening, when he felt better. The diarrhœa continued the second morning; and about 5, P. M., forty-nine hours after the diarrhœa set in, he had tenesmus and a mucous and bloody discharge. The disease continued seven days, and on the seventh or eighth day after he was attacked, No. 2 was seized with diarrhœa, which lasted one day, and then dysentery commenced, which terminated in three days. The day after No. 2 was attacked with dysentery, No. 3 was taken with diarrhœa, and after having it three days, dysentery set in, and continued four days. As soon as No. 3 was convalescent, on the fifth day of her dysentery, No. 4 was affected with diarrhœa, continuing one day, and running into dysentery, which was stopped on the second day. No. 5 was attacked the same afternoon that No. 4 was, with diarrhœa, lasting one day, and followed by dysentery continuing six days. No. 6 was seized on the fourth day of No. 5's dysentery, with severe diarrhœa, continuing two days: dysentery then set in, and—after continuing thirty-four days—terminated her life. Nos. 1, 5, and 6, suffered severely. No. 5, menstruation came on two days before the regular time; this appeared to aggravate the disease; the flow was quite menorrhagia; it ceased after continuing the usual time. During her illness, Dr. Rutherford and myself made a thorough examination of the premises and of the house, but could find no cause for the presence of the disease. The popular impression was that the disease was contagious, and induced by the use of peaches and other fruit.

Such are the facts. The writer will not draw any inferences, since he is attached to no theory nor to any particular mode of treatment. However, it has seemed to him that the name is very appropriate, the disease being something more than inflammation, something beyond "colonitis." He can well appreciate the significance of the word, dysentery.

### SECTION TENTH.

*On the Treatment.*

1. With opium or its preparations alone.

Case No. 4, after the dysentery had lasted a few hours, 40 drops of laudanum was given: this produced sleep, but on awakening, the discharges reappeared; 100 drops of laudanum were then given, and after a profound sleep of eight or nine hours, during which there appeared a copious perspiration, the patient awoke, feeling well, partook of a hearty meal, and returned to his accustomed avocations, with no further trouble from the complaint.

Case No. 11, after the disease had continued about six hours, 90 drops of laudanum were taken, profound sleep was produced, lasting eleven hours; perspiration occurred near the end of the slumber, and the patient awoke with a good appetite, and suffered no farther from the disease.

2. Opium or its preparations, combined with purgatives.

The remaining cases failed to be cut short by laudanum, but not enough probably was taken. Perfect rest and quiet were strictly enforced; the diet was reduced to gum-water. A dose of 5 grs. calomel was given, followed, if it did not operate, by castor oil; this generally brought on several evacuations of a black or dark green color. After this, calomel, gr. i, with opium, gr. $\frac{1}{4}$ to $\frac{1}{2}$, in the form of pill, was given every hour, unless the patient was asleep; sometimes sulph. morph., gr. $\frac{1}{16}$ to $\frac{1}{8}$ was substituted for the opium. These medicines were given till a mercurial impression was manifested, varying from one to three days. In the meantime the oil was given once every day, if there had been no fæcal discharges. It was noticeable, that as soon as the mercury began to affect the system, the tenesmus ceased, the mucous and bloody discharges were replaced by passages of a yellow color; refreshing sleep was soon obtained. The treatment subsequently was simple; the castor oil was continued every other day when no evacuation occurred, and at night the patient was given sulph. morph., gr. $\frac{1}{8}$ to $\frac{1}{3}$. As the dysenteric symptoms ceased, the diet was gradually improved. In Nos. 1 and 6, blisters were used over the abdomen. Enemas of opium were given in some of the cases, but with not much benefit to the patient. Every physician, with one exception, in the vicinity of Harrisburg, Pa., employs, invariably, calomel and some of preparation of opium, in dysentery. And, from their experience, and my own knowledge of this manner of medication, I must confess it, is eminently successful. Dr. Rutherford, of the above place, says, that the disease often terminates, without the use of any remedy, on the eighth or ninth day. The first six cases occurred near Harrisburg.

BUFFALO, June 30, 1856.

